# Seborrheic keratosis (Leser‐Trélat sign) in multiple myeloma signalling disease progression

**DOI:** 10.1002/jha2.88

**Published:** 2020-09-01

**Authors:** Christopher Jenkins, David Watson, Sewwandi Mendis, Glenda Hill, Lally De Soysa

**FIGURE 1:**
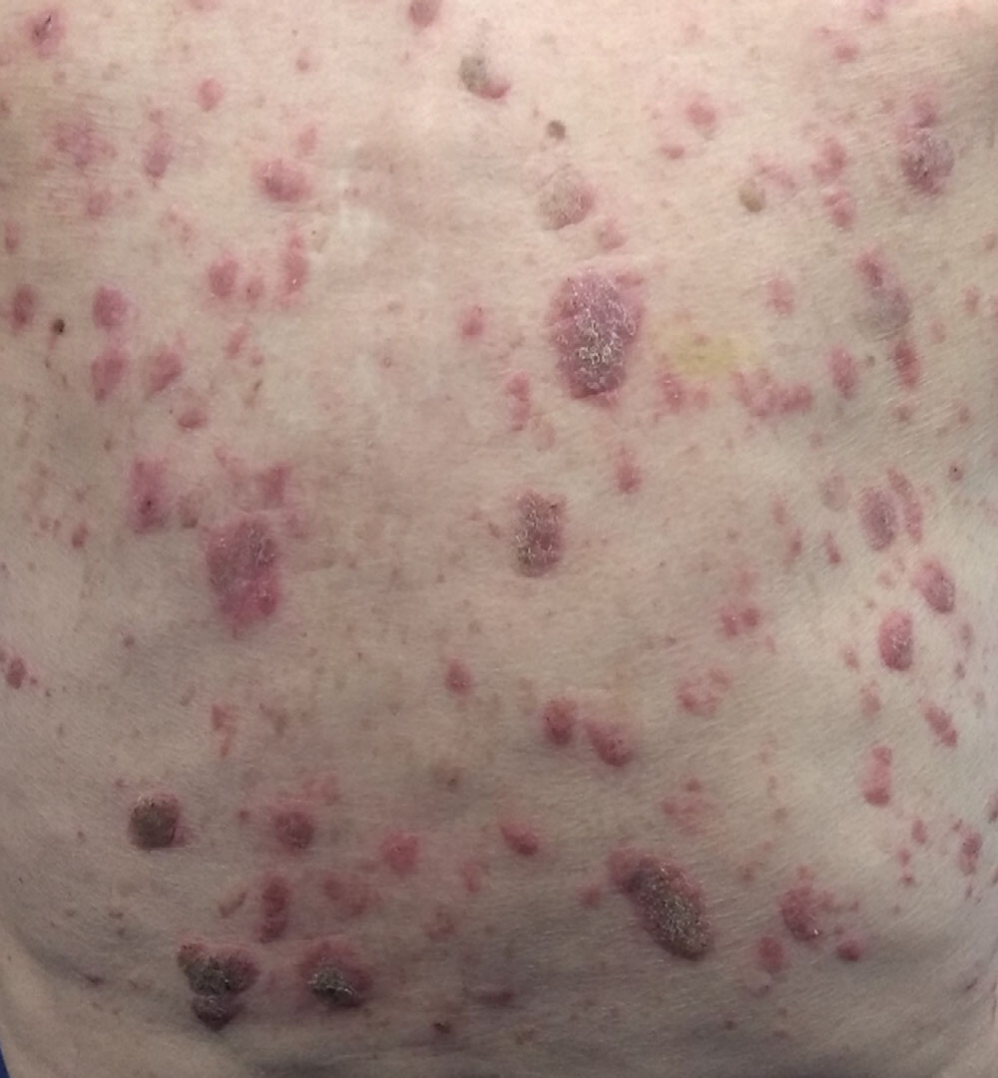
All new lesions on patients back that were not visible previously

A 90‐year‐old man with a diagnosis of immunoglobulin G Kappa (IgG Kappa) multiple myeloma presented with a 2 week history of new, intensely pruritic skin lesions on his back (Figure 1).

His hemoglobin concentration was 93 g/L, white blood cell count 3.9 × 10⁹/L, platelet count 214 × 10⁹/L, adjusted calcium 2.42 millimoles per liter, and estimated glomerular filtration rate 51 mL/min per 1.73 meters squared (baseline).  Paraprotein igG Kappa was 16.6 g/L, which had risen from 13.3 g/L 2 months previously.

A month prior to the skin lesions appearing, he had completed four cycles of melphalan and prednisolone chemotherapy. This was stopped because of patient fatigue.

Microscopy of a skin biopsy from a lesion on the patient's back showed benign and inflamed seborrhoeic keratosis, with no evidence of dysplasia or myeloma.  This is consistent with the sign of Leser‐Trélat.

Leser‐Trélat sign is a rare paraneoplastic phenomenon characterized by rapid onset, intensely pruritic, seborrheic keratosis that can vary in size and shape.  It is a poor prognostic sign, and should prompt further investigation for underlying malignancy if not previously known. Treatment of the underlying malignancy can resolve the lesions.

One cycle of bortezomib and dexamethasone completely resolved the lesions, with a dramatic effect on quality of life.

